# Numerical Solution for Fuzzy Time-Fractional Cancer Tumor Model with a Time-Dependent Net Killing Rate of Cancer Cells

**DOI:** 10.3390/ijerph20043766

**Published:** 2023-02-20

**Authors:** Hamzeh Zureigat, Mohammed Al-Smadi, Areen Al-Khateeb, Shrideh Al-Omari, Sharifah Alhazmi

**Affiliations:** 1 Department of Mathematics, Faculty of Science and Technology, Jadara University, Irbid 21110, Jordan; 2 College of Commerce and Business, Lusail University, Lusail 9717, Qatar; 3 Nonlinear Dynamics Research Center (NDRC), Ajman University, Ajman 20550, United Arab Emirates; 4 Department of Mathematics, Faculty of Science, Al-Balqa Applied University, Amman 11134, Jordan; 5 Mathematics Department, Al-Qunfudah University College, Umm Al-Qura University, Mecca 21955, Saudi Arabia

**Keywords:** cancer tumor models, fuzzy fractional diffusion equation, finite difference scheme, Caputo formula

## Abstract

**Simple Summary:**

One of the most recognized phenomena is the cancer tumor which uncontrollably grows in human cells and spreads over the other parts of the body. It spreads in many forms, including bone tumors, brain tumors, organ tumors, lung and pancreatic cancer tumors and others. This led to extensive research studying the cancer tumor model to follow up on the behavior of various cancer tumors in a body. In this paper, we discuss the impact of using a fuzzy time-fractional derivative in several cases of fuzzy initial conditions for the fuzzy time-fractional cancer tumor model. It was noted that there is a substantial need to study the fuzzy fractional cancer tumor model as it provides a comprehensive understanding of the behavior of the cancer tumor by taking into account several fuzzy cases in the initial condition of the proposed model.

**Abstract:**

A cancer tumor model is an important tool for studying the behavior of various cancer tumors. Recently, many fuzzy time-fractional diffusion equations have been employed to describe cancer tumor models in fuzzy conditions. In this paper, an explicit finite difference method has been developed and applied to solve a fuzzy time-fractional cancer tumor model. The impact of using the fuzzy time-fractional derivative has been examined under the double parametric form of fuzzy numbers rather than using classical time derivatives in fuzzy cancer tumor models. In addition, the stability of the proposed model has been investigated by applying the Fourier method, where the net killing rate of the cancer cells is only time-dependent, and the time-fractional derivative is Caputo’s derivative. Moreover, certain numerical experiments are discussed to examine the feasibility of the new approach and to check the related aspects. Over and above, certain needs in studying the fuzzy fractional cancer tumor model are detected to provide a better comprehensive understanding of the behavior of the tumor by utilizing several fuzzy cases on the initial conditions of the proposed model.

## 1. Introduction

The fractional partial differential equations act as significant tools for modeling many medical phenomena. One of the realized phenomena is the cancer tumor. A cancer tumor is a disease in which some of the body’s cells uncontrollably grow and spread over the other parts of the body. Cancer tumors have many types, including bone tumors, brain tumors, organ tumors, lung and pancreatic cancer tumors and others. Therefore, deficiencies in our understanding of cancer tumors have led to extensive research in this field. The problem has attracted not only biological and medical researchers but also mathematicians as well. Various approaches have been presented to discuss the growth and treatment responses of cancer tumors. Most of these approaches used statistical models such as the expectation-maximization approach or experimental methods [1,2,3,4]. In these studies, tumor decay or growth is discussed as a function of time. Laajala et al. [2] presented a statistical model to simulate the growth and treatment responses of tumor cells with time functions. Benzekry et al. [1] presented a model to discuss cancer cell proliferation based on a one-dimensional growth equation for different constant rates. The diffusion-based prototype model was proposed by Burgess et al. [5] to establish the interaction of growth rates and diffusion coefficients when the spherical cancer tumor has a therapy-dependent killing rate k and proliferation rate p. This prototype model was assumed in the following equation:

$$\frac{\partial u\left(x,t\right)}{\partial t}=D\frac{1}{r}\;\frac{\partial }{\partial r}\;\left(r^{2}\frac{\partial u\left(x,t\right)}{\partial r}\right)+p\;u\left(x,t\right)-k\;u\left(x,t\right),$$

where $u\left(x,t\right)$ is the concentration of cancer tumor cells at time t and position r, whereas the coefficient D represents the diffusivity coefficient. Later, Moyo and Leach [6] examined the one-dimensional type of this model by using the Lie symmetry method with a variable killing rate:

$$\frac{\partial u\left(x,t\right)}{\partial t}=\frac{\partial^{2}u\left(x,t\right)}{\partial x^{2}}-k\left(x,t\right)u\left(x,t\right),$$

where the net rate of the removal of the cancer tumor cells is described by K. The major contribution predicted that the net rate of removing the cancer tumor cells K could be a function of time and position, not necessarily dependent on constant or time only.

In the last few decades, the interest in fractional differential equations was expanded and has been applied to explain and develop some models of real-life phenomena which cannot be adequately explained by classical differential equations. The huge contribution of the fractional derivative has given a better and more comprehensive description of certain real-life problems. In the dynamics process of cancer tumors, significant properties take into consideration the mathematical modeling of the cancer tumor, such as the complex and irregular shapes and patterns, nonlinear growth dynamics, heterogeneity in cell distribution, proliferation, and self-similarity over different spatial and temporal scales. These properties can be quantified using fractal dimensions and other fractional derivatives of the parameters, allowing a better understanding of the tumor growth and progression and the potential improvement of diagnosis and treatment planning. Some of such problems were discussed in detail in the recent literature by many researchers [7,8,9,10].

The noteworthy application of the fractional derivative related to the cancer tumor model was given by Iomin (see [11]) on superdiffusion of cancer on a comb structure. This article shows that tumor growth is compatible with the fractional transport of cells. This indeed expands the attitude of fractional transport when the practical answer to the inquiry of how the neoplasm cells appear arbitrarily far from the main (primary) tumor in the case of the solid tumor. An analytical solution to the proposed model was presented in this paper. Meanwhile, Iyiola and Zaman [3] proposed a time-fractional cancer tumor model and discussed the essential requirement for a fractional order derivative as compared to the integer first-order time derivative. Three distinct cases of the net killing rate have been discussed. In the first case, the net killing rate is time-dependent only. In the second case, the net killing rate is the only space dependent. In the third case, the net killing rate depends on the concentration of the cancer tumor cells. It was also explained that the time-fractional derivative of order $\alpha \epsilon \;\left[0,1\right]$ would serve as an adequate model for the first case. However, in the third case, the time-fractional derivative of order $\alpha \epsilon \;\left[1,2\right]$ was recommended as the more efficient model.

In reality, the real phenomena are usually vague and contain uncertainties in the values of quantities of the governing model. Such uncertainty is known as stochastic uncertainty and is found in many fields, including manufacturing, medicine, engineering and others [12,13,14,15]. The fuzziness can arise in the data collection and measurement process. It can further appear in the process of calculating the boundary and initial conditions. Crisp quantities in the fractional diffusion equations, which are characterized as uncertain and imprecise, can be replaced by fuzzy quantities to reflect uncertainty and imprecision. This led to a fuzzy fractional diffusion equation. As discussed by many researchers, the cancer tumor model can be represented by a fractional diffusion equation [16,17,18,19,20,21,22,23,24,25,26,27,28]. However, in reality, the crisp quantities of the cancer tumor model are deemed uncertain. Hence, the fuzzy cancer tumor model is required to handle this problem. Very recently, Keshavarz et al. [29] discussed a solution to the fuzzy cancer tumor model using a certain analytical fuzzy transforms approach. The approach involved Caputo Hukuhara’s partial differentiability. Consequently, the impact of the fuzzy net killing rate of cells in the tumor was discussed and noted to achieve a better understanding of the model.

As the net killing rate of the cancer cells helps to observe the decay or growth of the cancer tumor, it would be worthwhile to investigate the model in different fuzzy cases and different fractional derivatives. This could help researchers to choose a particular treatment profile and provide a more practical and comprehensive description of the behavior of the cancer tumor. Therefore, the aim of this paper will conduct a study on solving the fuzzy time fractional tumor model when the net killing rate of the cancer cells is only time-dependent. In particular, a numerical explicit finite difference method is developed to solve the fuzzy time fractional tumor model in the double parametric form of fuzzy numbers. It also discussed the impact of using the fractional derivative instead of the integer derivative at different values of fractional order.

## 2. Time-Fractional Cancer Tumor Models in Fuzzy Environment

This section investigates the general form of the fuzzy time-fractional tumor model (FTFTM) discussed by fundamental concepts of the fuzzy theory and some related properties [30,31,32,33]. Consider the one-dimensional fuzzy time-fractional tumor model:

(1) $$\frac{\partial^{\alpha }\tilde{u}\left(x,t,\alpha \right)}{\partial^{\alpha }t}=\frac{\partial^{2}\tilde{u}\left(x,t\right)}{\partial x^{2}}-\tilde{k}\left(x,t\right)\;\tilde{u}\left(x,t\right)\;\;\;\;\;,\;0<\alpha \leq 1,\;\;\left(x,t\right)\epsilon \;\Omega =\left[0,L\right]\times \left[0,T\right]$$

with the initial and boundary conditions

$$\tilde{u}\left(x,0\right)=\tilde{f}\left(x\right),\;\tilde{u}\left(0,t\right)=\tilde{m}\left(0,t\right),\;\tilde{u}\left(l,t\right)=\tilde{n}\left(l,t\right),$$

where $\tilde{u}\left(x,t,\alpha \right)$ is the fuzzy concentration of the cancer tumor cells at time t and a fractional order α, $\tilde{k}\left(x,t\right)$ is the fuzzy net killing rate of the cancer cells of crisp variable t and x,$\;\frac{\partial^{\alpha }\tilde{u}\left(x,t,\alpha \right)}{\partial^{\alpha }t}$ is the fuzzy time fractional derivative of order α [34], $\frac{\partial^{2}\tilde{U}\left(x,t\right)}{\partial x^{2}}$ denotes the fuzzy partial Hukuhara derivatives with respect to x and $\tilde{u}\left(0,x\right)$ denotes the fuzzy initial condition. The boundary conditions in the fuzzy form are $\tilde{u}\left(0,t\right)$ and $\tilde{u}\left(l,0\right),$ which are equal to the fuzzy convex numbers $\tilde{m}$ and $\tilde{n},$ respectively. In addition, the fuzzy functions $\;k\text{ ˜}\left(x,t\right)$, $\tilde{f}\left(x\right)$ are defined as follows [35]

(2) $$\left\{\begin{array}{c} \overset{ˇ}{k}\left(x,t\right)=\tilde{\tau_{1}}\;s_{1}\left(x,t\right)\\ \tilde{f}\left(x\right)=\tilde{\tau_{2}}\;s_{2}\;\left(x\right) \end{array}\right.,\;\;\;$$

where $s_{1}\left(x,t\right)$ and $s_{2}\left(x\right)$ are the crisp functions of the crisp variable x and t, while ${\tilde{\tau }}_{1}$ and ${\tilde{\tau }}_{2}$ represent the fuzzy convex numbers. The FTFTM is defuzzified by using a single parametric approach of fuzzy numbers. The defuzzification of Equation (1) is given for all $r\in \left[0,1\right]$ as follows [35]:

(3) $${\left[\tilde{u}\left(x,t\right)\right]}_{r}=\underline{u}\left(x,t;r\right),\bar{u}\left(x,t;r\right)$$

(4) $${\left[\frac{\partial^{\alpha }\tilde{u}\left(x,t,\alpha \right)}{\partial^{\alpha }t}\right]}_{r}=\frac{\partial^{\alpha }\underline{u}\left(x,t,\alpha ;r\right)}{\partial^{\alpha }t},\frac{\partial^{\alpha }\bar{u}\left(x,t,\alpha ;r\right)}{\partial^{\alpha }t}\;\;$$

(5) $${\left[\frac{\partial^{2}\tilde{u}\left(x,t\right)}{\partial x^{2}}\right]}_{r}=\frac{\partial^{2}\underline{u}\left(x,t;r\right)}{\partial x^{2}},\;\;\;\frac{\partial^{2}\bar{u}\left(x,t;r\right)}{\partial x^{2}}$$

(6) $${\left[\;k\text{ ˜}\left(x,t\right)\right]}_{r}=\;k\text{ \_}\left(x,t;r\right),\;k\text{ ¯}\left(x,t;r\right)$$

(7) $${\left[\tilde{u}\left(x,0\right)\right]}_{r}=\underline{u}\left(x,0;r\right),\bar{u}\left(x,0;r\right)$$

(8) $${\left[\tilde{u}\left(0,t\right)\right]}_{r}=\underline{u}\left(0,t;r\right),\bar{u}\left(0,t;r\right)$$

(9) $${\left[\tilde{u}\left(l,t\right)\right]}_{r}=\underline{u}\left(l,t;r\right),\bar{u}\left(l,t;r\right)$$

(10) $${\left[\tilde{f}\left(x\right)\right]}_{r}=\underline{f}\left(x;r\right),\bar{f}\left(x;r\right)$$

(11) $$\left\{\begin{array}{c} {\left[\tilde{m}\right]}_{r}=\underline{m}\left(t;r\right),\bar{m}\left(t;r\right)\\ {\left[\tilde{n}\right]}_{r}=\underline{n}\left(l;r\right),\bar{n}\left(l;r\right) \end{array}\right.\;$$

where

(12) $$\left\{\begin{array}{c} {\left[\;k\text{ ˜}\left(x,t\right)\right]}_{r}=\left[\underline{\tau }{\left(r\right)}_{1},{\bar{\tau }}_{1}\left(r\right)\right]s_{1}\left(x,t\right)\;\\ {\left[\tilde{f_{1}}\left(x\right)\right]}_{r}=\left[\underline{\tau }{\left(r\right)}_{2},{\bar{\tau }}_{2}\left(r\right)\right]s_{2}\left(x\right) \end{array}\right.\;\;\;\;$$

The membership function is defined by using the fuzzy extension principle [35]

(13) $$\left\{\begin{array}{c} \underline{u}\left(x,t;r\right)=min\left\{\tilde{u}\left(\tilde{\mu }\left(r\right),t)\right)|\tilde{\mu }\left(r\right)\in \tilde{u}\left(x,t;r\right)\right\}\\ \bar{u}\left(x,t;r\right)=max\left\{\tilde{u}\left(\tilde{\mu }\left(r\right),t\right)|\tilde{\mu }\left(r\right)\in \tilde{u}\left(x,t;r\right)\right\} \end{array}\right.$$

As per the singular parametric form, we may write Equation (1) as follows

(14) $$\left[\frac{\partial^{\alpha }\underline{u}\left(x,t,\alpha ;r\right)}{\partial^{\alpha }t}\;,\;\frac{\partial^{\alpha }\bar{u}\left(x,t,\alpha ;r\right)}{\partial^{\alpha }t}\right]=\left[\frac{\partial^{2}\underline{u}\left(x,t;r\right)}{\partial x^{2}}\;,\;\frac{\partial^{2}\bar{u}\left(x,t;r\right)}{\partial x^{2}}\right]-\left[\;\underline{k}\left(x,t;r\right)\underline{u}\left(x,t;r\right)\;,\bar{k}\left(x,t;r\right)\bar{u}\left(x,t;r\right)\right],\;$$

Equipped with the fuzzy initial and boundary conditions

$$\left[\;\underline{u}\left(x,0;r\right),\bar{u}\left(x,0;r\right)\right]=\left[\underline{f}\left(x,t;r\right),\bar{f}\left(x,t;r\right)\right],$$

$$\left[\;\underline{u}\left(0,t;r\right),\bar{u}\left(0,t;r\right)\right]=\left[\underline{m}\left(0,t;r\right),\bar{m}\left(0,t;r\right)\right],$$

$$\left[\;\underline{u}\left(l,t;r\right),\bar{u}\left(l,t;r\right)\right]=\left[\underline{n}\left(l,t;r\right),\bar{n}\left(l,t;r\right)\right]$$

Now, based on the given approach, the double parametric form [36], we rewrite Equation (14) as follows

$$\begin{array}{cc} \left\{\;\beta \;\left(\frac{\partial^{\alpha }\bar{u}\left(x,t,\alpha ;r\right)}{\partial^{\alpha }t}\right)\right. & -\left.\left(\frac{\partial^{\alpha }\underline{u}\left(x,t,\alpha ;r\right)}{\partial^{\alpha }t}\;\right)+\frac{\partial^{\alpha }\underline{u}\left(x,t,\alpha ;r\right)}{\partial^{\alpha }t}\right\}\\ = & \left\{\;\beta \;\left(\frac{\partial^{2}\bar{u}\left(x,t;r\right)}{\partial x^{2}}-\frac{\partial^{2}\underline{u}\left(x,t;r\right)}{\partial x^{2}}\right)+\frac{\partial^{2}\underline{u}\left(x,t;r\right)}{\partial x^{2}}\right\}\\ - & \left\{\;\beta \;\left(\bar{k}\left(x,t,r\right)-\underline{k}\left(x,t,r\right)\right)+\underline{k}\left(x,t,r\right)\right\}\;\;\left\{\;\beta \;\left(\bar{u}\left(x,t;r\right)-\underline{u}\left(x,t;r\right)\right)+\underline{u}\left(x,t;r\right)\right\}, \end{array}$$

subject to the fuzzy initial and boundary conditions

$$\left\{\;\beta \;\left(\bar{u}\left(x,0;r\right)-\underline{u}\left(x,0;r\right)\right)+\underline{u}\left(x,0;r\right)\right\}=\left\{\;\beta \;\left(\bar{f}\left(x;r\right)-\underline{f}\left(x;r\right)\right)+\underline{f}\left(x;r\right)\right\}\;,$$

$$\left\{\;\beta \;\left(\bar{u}\left(0,t;r\right)-\underline{u}\left(0,t;r\right)\right)+\underline{u}\left(0,t;r\right)\right\}=\left\{\;\beta \;\left(\bar{m}\left(t;r\right)-\underline{m}\left(t;r\right)\right)+\underline{m}\left(t;r\right)\right\},$$

$$\left\{\;\beta \;\left(\bar{u}\left(l,t;r\right)-\underline{u}\left(l,t;r\right)\right)+\underline{u}\left(l,t;r\right)\right\}=\left\{\;\beta \;\left(\bar{n}\left(t;r\right)-\underline{n}\left(t;r\right)\right)+\underline{n}\left(t;r\right)\right\},$$

where $\beta \in \left[0,1\right]$. Now, we write

$$\frac{\partial^{\alpha }\tilde{u}\left(x,t,\beta \right)}{\partial^{\alpha }t}=\left\{\;\beta \;\left(\frac{\partial^{\alpha }\bar{u}\left(x,t,\alpha ;r\right)}{\partial^{\alpha }t}-\frac{\partial^{\alpha }\underline{u}\left(x,t,\alpha ;r\right)}{\partial^{\alpha }t}\;\right)+\frac{\partial^{\alpha }\underline{u}\left(x,t,\alpha ;r\right)}{\partial^{\alpha }t}\right\},$$

$$\frac{\partial^{2}\tilde{u}\left(x,t,\beta \right)}{\partial x^{2}}=\left\{\;\beta \;\left(\frac{\partial^{2}\bar{u}\left(x,t;r\right)}{\partial x^{2}}-\frac{\partial^{2}\underline{u}\left(x,t;r\right)}{\partial x^{2}}\right)+\frac{\partial^{2}\underline{u}\left(x,t;r\right)}{\partial x^{2}}\right\},$$

$$\tilde{k}\left(x,t;r,\beta \right)=\left\{\;\beta \;\left(\bar{k}\left(x,t;r\right)-\underline{k}\left(x,t;r\right)\right)+\underline{k}\left(x,t;r\right)\right\},$$

$$\tilde{u}\left(x,t;r,\beta \right)=\left\{\;\beta \;\left(\bar{u}\left(x,t;r\right)-\underline{u}\left(x,t;r\right)\right)+\underline{u}\left(x,t;r\right)\right\},$$

$$\tilde{u}\left(x,0,r,\beta \right)=\left\{\;\beta \;\left(\bar{u}\left(x,0;r\right)-\underline{u}\left(x,0;r\right)\right)+\underline{u}\left(x,0;r\right)\right\},$$

$$\tilde{u}\left(x,0,r,\beta \right)=\left\{\;\beta \;\left(\bar{u}\left(x,0;r\right)-\underline{u}\left(x,0;r\right)\right)+\underline{u}\left(x,0;r\right)\right\},\tilde{f}\left(x;r,\beta \right)=\left\{\;\beta \;\left(\bar{f}\left(x;r\right)-\underline{f}\left(x;r\right)\right)+\underline{f}\left(x;r\right)\right\},$$

$$\;u\text{ ˜}\left(x,0,r,\beta \right)=\left\{\;\beta \;\left(\;u\text{ ¯}\left(x,0;r\right)-\;u\text{ \_}\left(x,0;r\right)\right)+\;u\text{ \_}\left(x,0;r\right)\right\},\;f\text{ ˜}\left(x;r,\beta \right)=\left\{\;\beta \;\left(\;f\text{ ¯}\left(x;r\right)-\;f\text{ \_}\left(x;r\right)\right)+\;f\text{ \_}\left(x;r\right)\right\},$$

$$\tilde{u}\left(0,t,r,\beta \right)=\left\{\;\beta \;\left(\underline{u}\left(0,t;r\right)-\bar{u}\left(0,t;r\right)\right)+\underline{u}\left(0,t;r\right)\right\},$$

$$\tilde{m}\left(t,r,\beta \right)=\left\{\;\beta \;\left(\bar{m}\left(t;r\right)-\underline{m}\left(t;r\right)\right)+\underline{m}\left(t;r\right)\right\}$$

$$\tilde{u}\left(l,t,r,\beta \right)=\left\{\;\beta \;\left(\underline{u}\left(l,t;r\right)-\bar{u}\left(l,t;r\right)\right)+\underline{u}\left(l,t;r\right)\right\},$$

$$\tilde{n}\left(t,r,\beta \right)=\left\{\;\beta \;\left(\bar{n}\left(t;r\right)-\underline{n}\left(t;r\right)\right)+\underline{n}\left(t;r\right)\right\}.$$

Then, substituting the above equations into Equation (14) yields the general form of the time-fractional cancer tumor model

$$\frac{\partial^{\alpha }\tilde{u}\left(x,t,\alpha ;r,\beta \right)}{\partial^{\alpha }t}=\frac{\partial^{2}\tilde{u}\left(x,t;r,\beta \right)}{\partial x^{2}}-\tilde{k}\left(x,t;r,\beta \right)\;\;\tilde{u}\left(x,t;r,\beta \right)\;,\;\;\;0\leq r\leq 1,0\leq \beta \leq 1,$$

(15) $$\tilde{u}\left(x,0;r,\beta \right)=\tilde{f}\left(x;r,\beta \right)\;,\;\tilde{u}\left(0,t,\beta \right)=\tilde{m}\;,\;\tilde{u}\left(l,t,\beta \right)=\tilde{n}.$$

To obtain the lower and upper bounds of the solutions, respectively, we assume β=0 and β=1 , which may be presented as $\tilde{u}\left(x,t;r,0\right)=\underline{u}\left(x,t;r\right)\;\mathrm{and}\;\tilde{u}\left(x,t;r,1\right)=\bar{u}\left(x,t;r\right).\;$

## 3. Explicit Finite Difference Scheme for Solving Fuzzy Cancer Tumor Models

In this section, an explicit finite difference method is implemented in Caputo sense for time-fractional derivative and central difference approximation at time level n, for second order space derivative, to solve the fuzzy time-fractional tumor model under the double parametric form of fuzzy numbers. The time-fractional derivative in Equation (15) is discretized using the Caputo formula as [37]

(16) $$\frac{\partial^{\alpha }\tilde{u}\left(x,t,\alpha \right)}{\partial^{\alpha }t}=\frac{\Delta t^{-\alpha }}{\Gamma \left(2-\alpha \right)}[u_{i}^{n+1}\left(x,t;r,\beta \right)-u_{i}^{n}\left(x,t;r,\beta \right)+\overset{n}{\underset{j=1}{\sum }}b_{j}\;\left(u_{i}^{n+1-j}\left(x,t;r,\beta \right)-u_{i}^{n-j}\left(x,t;r,\beta \right)\right)]+O\left(\Delta t\right),$$

where $b_{j}={\left(j+1\right)}^{1-\alpha }-{\left(j\right)}^{1-\alpha }\;,\;j=1,2,\ldots$.

The central difference approximation at time level n is used to discretize the second partial derivative as follows

(17) $$\frac{\partial^{2}\tilde{u}\left(x,t;r,\beta \right)}{\partial x^{2}}=\frac{{\tilde{u}}_{i+1}^{n}\left(x,t;r,\beta \right)-2{\tilde{u}}_{i}^{n}\left(x,t;r,\beta \right)+{\tilde{u}}_{i-1}^{n}\left(x,t;r,\beta \right)}{\Delta x^{2}}.$$

We substitute Equations (16) and (17) into Equation (15) to obtain

(18) $$\frac{\Delta t^{-\alpha }}{\Gamma \left(2-\alpha \right)}\left[u_{i}^{n+1}\left(x,t;r,\beta \right)-u_{i}^{n}\left(x,t;r,\beta \right)+\overset{n}{\underset{j=1}{\sum }}b_{j}\;\left(u_{i}^{n+1-j}\left(x,t;r,\beta \right)-u_{i}^{n-j}\left(x,t;r,\beta \right)\right)\right]=\;\frac{{\tilde{u}}_{i+1}^{n}\left(x,t;r,\beta \right)-2{\tilde{u}}_{i}^{n}\left(x,t;r,\beta \right)+{\tilde{u}}_{i-1}^{n}\left(x,t;r,\beta \right)}{\Delta x^{2}}-\tilde{k}\left(x,t;r,\beta \right)\;{\tilde{u}}_{i}^{n}\left(x,t;r,\beta \right).$$

By letting $\tilde{s}\left(r,\beta \right)=\frac{\Delta t^{\alpha }\;\Gamma \left(2-\alpha \right)}{\Delta x^{2}}$ and $r,\beta \in \left[0,1\right]$, and employing Equation (18), we get

(19) $${\tilde{u}}_{i}^{n+1}\left(x,t;r,\beta \right)=s\;\left[{\tilde{u}}_{i+1}^{n}\left(x,t;r,\beta \right)+{\tilde{u}}_{i-1}^{n}\left(x,t;r,\beta \right)\right]+\left(1-2s\right){\tilde{u}}_{i}^{n}\left(x,t;r,\beta \right)\;-\Delta t^{\alpha }\;\Gamma \left(2-\alpha \right)\;\tilde{k}\left(x,t;r,\beta \right)\;{\tilde{u}}_{i}^{n}\left(x,t;r,\beta \right)-\overset{n}{\underset{j=1}{\sum }}b_{j}\;\left(u_{i}^{n+1-j}\left(x,t;r,\beta \right)-u_{i}^{n-j}\left(x,t;r,\beta \right)\right).\;$$

For each spatial grid point, the equations in (12) are evaluated to yield linear equations. At the end of each time level, a system of linear equations is established to obtain the values $\tilde{u}\left(x,t,\alpha ,\beta \right)$ for the particular time level.

## 4. Stability Analysis

It is first assumed that the discretization of the initial condition yields the fuzzy error ${\tilde{\varepsilon }}_{i}^{0}$. Let ${\tilde{u}}_{i}^{0}=\overset{´}{\;{\tilde{u}}_{i}^{0}}-{\tilde{\varepsilon }}_{i}^{0}$, ${\tilde{u}}_{i}^{n}$ and $\overset{´}{{\tilde{u}}_{i}^{n}}$ be the fuzzy numerical solutions of the scheme of Equation (19), with respect to the initial data’s ${\tilde{f}}_{i}^{0}$ and $\overset{´}{\;{\tilde{f}}_{i}^{0}},$ respectively. Let ${\left[{\tilde{u}}_{i}^{n}\left(x,t;\alpha \right)\right]}_{r}=\beta \left[\bar{u}\left(r\right)-\underline{u}\left(r\right)\right]+\underline{u}\left(r\right)$, where $\beta ,r\in \left[0,1\right]$. Then, the fuzzy error bound is defined as

(20) $${\left[{\tilde{\varepsilon }}_{i}^{n}\right]}_{r}={\left[\overset{´}{{\tilde{u}}_{i}^{n}}-{\tilde{u}}_{i}^{n}\right]}_{r}\;,\;\;\;\;n=1,2,\;\ldots \ldots N-1,\;\;i=1,2,\ldots ,M-1$$

Now, based on the approach used in [38], Equation (19) can be rewritten as follows

(21) $${\tilde{u}}_{i}^{n+1}=s\;{\tilde{u}}_{i+1}^{n}+\left(1-2s-\Delta t^{\alpha }\;\Gamma \left(2-\alpha \right)\;\tilde{k}\left(x,t\right)-b_{1}\right){\tilde{u}}_{i}^{n}+s\;{\tilde{u}}_{i-1}^{n}-\overset{n-1}{\underset{j=1}{\sum }}(b_{j+1}-b_{j})\left({\tilde{u}}_{i}^{n-j}\right)+b_{n}{\tilde{u}}_{i}^{0}\;.\;$$

From Equation (21), we infer $\Delta t^{\alpha }\;\Gamma \left(2-\alpha \right)\;\tilde{k}\left(x,t\right)=s\Delta x^{2}\;\tilde{k}\left(x,t\right).$ Therefore, we rewrite the fuzzy round-off error for Equation (21) as

(22) $${\tilde{\varepsilon }}_{i}^{n+1}=s\;{\tilde{\varepsilon }}_{i+1}^{n}+\left(1-2s-s\;\Delta x^{2}\;\;\tilde{k}\left(x,t\right)-b_{1}\right){\tilde{\varepsilon }}_{i}^{n}+s{\tilde{\varepsilon }}_{i-1}^{n}-\overset{n-1}{\underset{j=1}{\sum }}(b_{j+1}-b_{j})\left({\tilde{\varepsilon }}_{i}^{n-j}\right)+b_{n}{\tilde{\varepsilon }}_{i}^{0}\;.$$

Assume ${\tilde{\varepsilon }}_{0}^{n}={\tilde{\varepsilon }}_{X}^{n}=0,\;n=1,2,\ldots .,N-1$ and ${\tilde{\varepsilon }}_{i}^{n}=[{\tilde{\varepsilon }}_{1}^{n},{\tilde{\varepsilon }}_{2}^{n},\ldots \ldots ,{\tilde{\varepsilon }}_{X-1}^{n}$]. Then, introduce the fuzzy norm

(23) $$\;\|{\tilde{\varepsilon }}^{n}\|{}_{2}=\sqrt{\sum_{i=1}^{X-1}h\;{\left|{\tilde{\varepsilon }}_{i}^{n}\right|}^{2}},$$

which gives

(24) $$\|{\tilde{\varepsilon }}^{n}\|{}_{2}^{2}=\overset{\infty }{\underset{i=-\infty }{\sum }}\;{\left|{\tilde{\lambda }}^{n}\right|}^{2}$$

Hence, ${\tilde{\varepsilon }}_{i}^{n}$ may alternatively be expressed as

(25) $${\tilde{\varepsilon }}_{i}^{n}={\tilde{\lambda }}^{n}\;e^{\sqrt{-\theta_{i}}}\;,\;\;\mathrm{where}\;{\tilde{\theta }}_{i}=qih$$

Therefore, by substituting Equation (25) into Equation (22), we derive

(26) $${\tilde{\lambda }}^{n+1}\;e^{\sqrt{-\theta_{i}}}=s\;{\tilde{\lambda }}^{n}\;e^{\sqrt{-\theta_{i+1}}}+\left(1-2s-s\;\Delta x^{2}\;\;\tilde{k}\left(x,t\right)-b_{1}\right){\tilde{\lambda }}^{n}\;e^{\sqrt{-\theta_{i}}}+s\;{\tilde{\lambda }}^{n}\;e^{\sqrt{-\theta_{i-1}}}\;-\overset{n-1}{\underset{j=1}{\sum }}(b_{j+1}-b_{j}){\tilde{\lambda }}^{n-j}\;e^{\sqrt{-\theta_{i}}}+b_{n}{\tilde{\lambda }}^{0}\;e^{\sqrt{-\theta_{i}}}\;.$$

Dividing Equation (26) by $\;e^{\sqrt{-\theta i}}$ reveals

(27) $${\tilde{\lambda }}^{n+1}=\left[\left(1-2s-s\;\Delta x^{2}\;\;\tilde{k}\left(x,t\right)-b_{1}\right)+s(\;e^{\sqrt{-\theta_{i}}}+e^{-\sqrt{-\theta_{i}}}\right)]{\tilde{\lambda }}^{n}-\overset{n-1}{\underset{j=1}{\sum }}(b_{j+1}-b_{j}){\tilde{\lambda }}^{n-j}+b_{n}{\tilde{\lambda }}^{0}\;,$$

(28) $${\tilde{\lambda }}^{n+1}=\left[\left(1-2s-s\;\Delta x^{2}\;\;\tilde{k}\left(x,t\right)-b_{1}\right)+s\left(2-4\sin^{2}\left(\frac{\theta }{2}\right)\right)\right]{\tilde{\lambda }}^{n}-\overset{n-1}{\underset{j=1}{\sum }}(b_{j+1}-b_{j}){\tilde{\lambda }}^{n-j}+b_{n}{\tilde{\lambda }}^{0}\;.\;$$

Hence, simplifying Equation (28) yields

(29) $${\tilde{\lambda }}^{n+1}=\left[1-s\;\Delta x^{2}\;\;\tilde{k}\left(x,t\right)-b_{1}-4s\sin^{2}\left(\frac{\theta }{2}\right)\right)]{\tilde{\lambda }}^{n}-\overset{n-1}{\underset{j=1}{\sum }}(b_{j+1}-b_{j}){\tilde{\lambda }}^{n-j}+b_{n}{\tilde{\lambda }}^{0}\;.$$

**Proposition** **1.** *If*${\tilde{\lambda }}^{n}$*is the fuzzy solution of Equation (21) and*$s\leq \;\frac{1}{4}\;\left(1-\Delta t^{\alpha }\;\Gamma \left(2-\alpha \right)\;\tilde{k}\left(x,t\right)-b_{1}\right),$*then*$\left|{\tilde{\lambda }}^{n}\right|\leq \left|{\tilde{\lambda }}^{0}\right|$.

**Proof.**  For =0, from Equation (29), we have

$${\tilde{\lambda }}^{1}=\left[1-s\;\Delta x^{2}\;\;\tilde{k}\left(x,t\right)-4s\sin^{2}\left(\frac{\theta }{2}\right)\right]{\tilde{\lambda }}^{0}.$$

In view of the facts that $s\leq \;\frac{1}{4}\;\mathrm{and}\;\sin^{2}\left(\frac{\theta }{2}\right)\geq 0$, we have

$$\left|{\tilde{\lambda }}^{1}\right|\leq \left|{\tilde{\lambda }}^{0}\right|.\;$$

This completes the proof of our result. □

In what follows, we may assume that $\left|{\tilde{\lambda }}^{m}\right|\leq \left|{\tilde{\lambda }}^{0}\right|\;,\;m=1,\;2\;,\;3\;,\;\ldots ,n-1$. Therefore, we state without proof the following lemma.

**Lemma** **1.** *The coefficients* $\;b_{j}={\left(j+1\right)}^{1-\alpha }-{\left(j\right)}^{1-\alpha }\;,\;j=1,2,\ldots$*satisfy the following conditions* [38]

(1)
$0<b_{j}\leq 1\;,\;j=1\;,\;2\;,\;3,\ldots$ *,*
(2)
$\;b_{j}>b_{j+1}\;j=1,2\;,\;3,\;\ldots ,$

(3)
$\overset{n-1}{\underset{j=1}{\sum }}(b_{j+1}-b_{j})=1-b_{n}\;.$

Consequently, from Lemma 1 and Equation (29), we infer that

$$\left|{\tilde{\lambda }}^{n+1}\right|\leq \;\left[1-s\;\Delta x^{2}\;\;\tilde{k}\left(x,t\right)-b_{1}-4p\sin^{2}\left(\frac{\theta }{2}\right)\right)]\left|{\tilde{\lambda }}^{n}\right|-\overset{n-1}{\underset{j=1}{\sum }}(b_{j+1}-b_{j})\left|{\tilde{\lambda }}^{n-j}\right|+b_{n}\left|{\tilde{\lambda }}^{0}\right|\;,$$

$$\left|{\tilde{\lambda }}^{n+1}\right|\leq \;\left[1-s\;\Delta x^{2}\;\;\tilde{k}\left(x,t\right)-b_{1}-4s\sin^{2}\left(\frac{\theta }{2}\right)\right)-\left(b_{n}-b_{1}\right)+b_{n}]\left|{\tilde{\lambda }}^{0}\right|\leq \;\left|{\tilde{\lambda }}^{0}\right|.$$

**Theorem** **1.** *The explicit finite difference scheme is* *stable under the condition*

$$s\leq \;\frac{1}{4}-s\;\Delta x^{2}\;\;\tilde{k}\left(x,t\right)$$

**Proof.**  From the formula given by Proposition 1, it can be established that

$$\|{\tilde{\varepsilon }}^{n}\|{}_{2}\leq \|{\tilde{\varepsilon }}^{0}\|{}_{2}\;,\;\;\;\;\;n=1,2,\ldots .,N-1\;,$$

which means that the explicit finite difference scheme is stable under the condition

$$s\leq \;\frac{1}{4}-s\;\Delta x^{2}\;\tilde{k}\left(x,t\right).□$$

## 5. Numerical Experiment and Discussion

Consider the fuzzy time-fractional tumor model when the net killing rate of the cancer cells is only time-dependent [4]

(30) $$\frac{\partial^{\alpha }\tilde{u}\left(x,t,\alpha \right)}{\partial^{\alpha }t}=\frac{\partial^{2}\tilde{u}\left(x,t\right)}{\partial x^{2}}-t^{2}\;\tilde{u}\left(x,t\right)\;\;,\;0<\alpha \leq 1$$.

Assume the fuzzy initial condition $\mathrm{is}\;\tilde{u}\left(x,0\right)=\tilde{\emptyset }\left(r,\beta \right)\;e^{kx}$, where

$$\tilde{\emptyset }\left(r,\beta \right)=\beta \;\left[\left(1-r\right)-\left(r-1\right)\right]+\left(r-1\right),\;\;r\;\mathrm{and}\;\beta \in \left[0,1\right].$$

Then, the exact fuzzy solution of Equation (30) was given in [4] as

$$\tilde{u}\left(x,t,\alpha \right)=\tilde{\emptyset }\left(r,\beta \right)\left(e^{kx}+e^{kx}k^{2}\;\frac{t^{\alpha }}{\Gamma \left(1+\alpha \right)}+\frac{t^{2\alpha }}{\Gamma \left(1+2\alpha \right)}\;e^{kx}k^{4}\right).$$

Therefore, the absolute error of the solution of Equation (30) can be defined as

$${\left[\tilde{E}\right]}_{r}=\left|\tilde{U}\left(t,x;r\right)-\tilde{u}\left(t,x;r\right)\right|.$$

At $\Delta x=0.5\;\mathrm{and}\;\Delta t=0.01=0.1\;,\;\tilde{s}\left(r,\beta \right)=\frac{\Delta t^{\alpha }\;\Gamma \left(2-\alpha \right)}{\Delta x^{2}}$, we have the following results.

**Figure 1:**
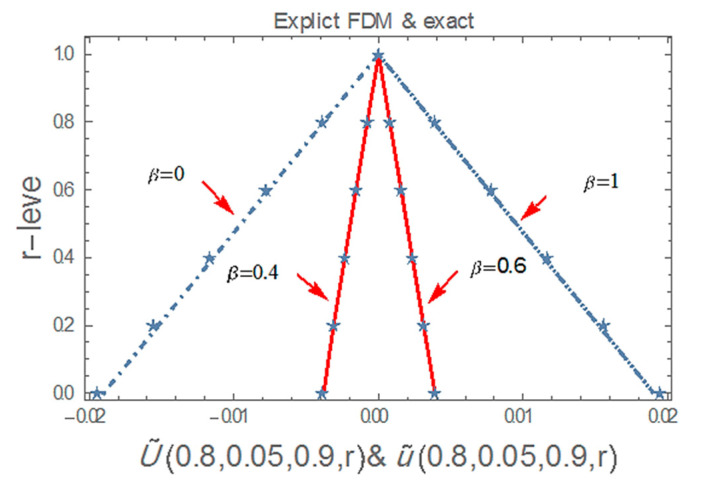
The fuzzy numerical solution of Equation (30), by using explicit finite difference at α=0.9, x=4, t=0.05 for all $r,\beta \in \left[0,1\right]$.

**Figure 2:**
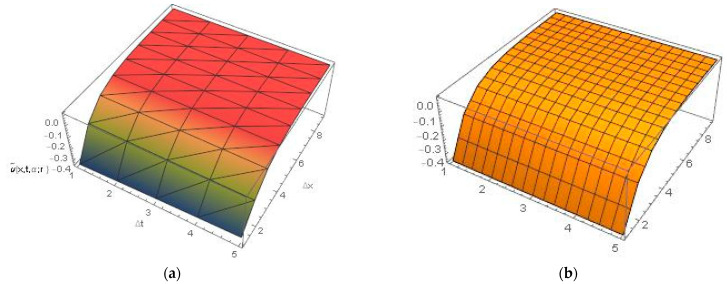
(**a**) Lower numerical solution of Equation (30), by explicit finite difference, (**b**) Lower exact solution at t=0.05,x=4 , α=0.9 and β=0  for all $r\in \left[0,1\right]$.

**Figure 3:**
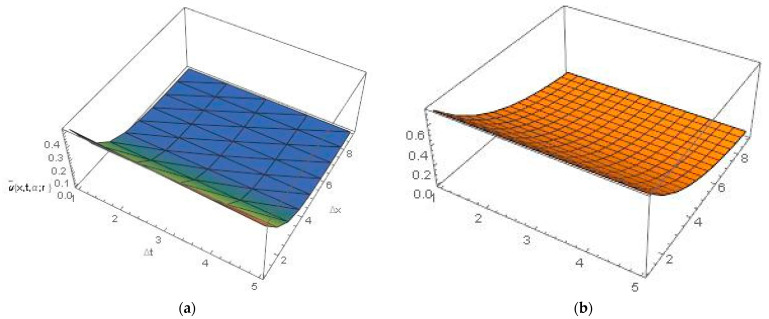
(**a**) Upper numerical solution of Equation (30), by explicit finite difference, (**b**) Upper exact solution at t=0.05,x=4 , α=0.9 and β=1  for all $r\in \left[0,1\right]$.

Table 1, Figure 1, Figure 2 and Figure 3 shows that the explicit finite difference methods have a good agreement with the exact solution at x=4,t=0.05, $\alpha =0.9\;,\;r,\beta \in \left[0,1\right]$ and satisfy the properties of the fuzzy numbers by considering triangular fuzzy number shape. Also, as we can see in Table 1 and Figure 1, we can be more accurate by decreasing the value of r. The obtained numerical results are the more precise solution at the points which are close to the inflection value β=0.5, where the inflection value is the value that the fuzzy solutions turn from the lower solutions to the upper solutions.

**Figure 4:**
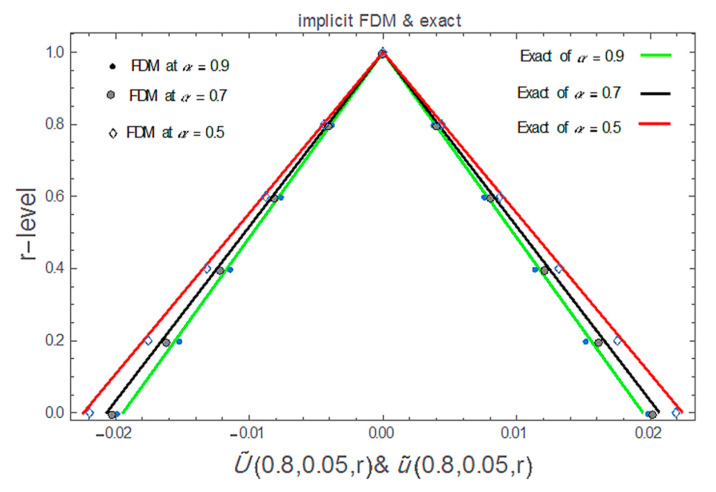
Exact and explicit FDM of the solution of Equation (30) at different values of α for all $r\in \left[0,1\right].$.

**Figure 5:**
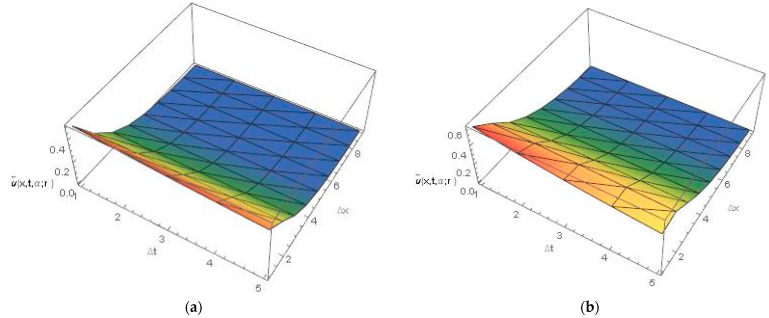
(**a**,**b**) fuzzy numerical solution of Equation (30), at different time and space steps at α=0.9 for all $r\in \left[0,1\right]$.

Figure 2 and Figure 3 represent the 3D graphics of the numerical explicit finite difference and the exact solution. They also show that the net killing rate of cancer cells increases with time. Furthermore, as we can see in Figure 4, the explicit finite difference solution agrees with the exact solution for different values of α. The comparison of the numerical and exact solutions when α=0.5 , 0.7 and 0.9 show that the numerical solution is more accurate when the value of α tends to 1. Finally, Figure 5 shows that the proposed scheme is validated at different time and space steps taking into account the stability condition for the proposed approach, which is discussed in Section 4.

From all of the above, it is clear that using the fuzzy fractional cancer tumor model is more practical and feasible as compared to the crisp fractional cancer tumor model since it improves accuracy in predicting the growth and spread of cancer tumors, see [22,39,40,41,42,43]. The approach also handles the uncertainty and ambiguity in the data, such as the uncertainty in the initial condition Equation (30), which is discussed in the presented example, and by the ability to capture nonlinear and non-instantaneous behavior of the tumor growth.

## 6. Conclusions

In this paper, the impact of using a fuzzy time-fractional derivative instead of the classical time derivative in the fuzzy cancer tumor model is discussed by taking into account different values of fractional derivatives under several cases of fuzzy initial conditions of the fuzzy time-fractional cancer tumor model. An explicit finite difference method is developed and applied to numerically solve the fuzzy time-fractional cancer tumor model. As related to the net killing rate, we focus on the case when the net killing rate of the cancer cells only depends on time. The time-fractional derivative is replaced by employing Caputo’s definition. The Fourier method was applied to investigate the stability of the numerical approach. Finally, a numerical example has been presented to examine the feasibility of the proposed approach and to check certain related aspects. It was found that there is a substantial need to study the fuzzy fractional cancer tumor model since it provides a comprehensive understanding of the behavior of the cancer tumor by taking into account covering several fuzzy cases in the initial condition of the proposed model, which could help researchers to choose a particular treatment profile. The presented scheme may be extended to study the connection between the fuzzy fractional cancer model and the bifurcation analysis of the fractional tumor models. This study will be investigated in detail at a later stage.

## Figures and Tables

**Table 1:** Fuzzy numerical solution of Equation (30), by explicit finite difference scheme at t=0.05,x=4 and α=0.9 for all $r,\beta \in \left[0,1\right]$.

β	r	$\tilde{u}\left(4,0.05;r,\beta \right)$	$\tilde{E}\left(4\;,0.05;r,\beta \right)\mathrm{Abs}\;\mathrm{Error}$	β	r	$\tilde{u}\left(4,0.05;r,\beta \right)$	$\tilde{E}\left(4,0.05;r,\beta \right)\mathrm{Abs}\;\mathrm{Error}$
Lower solution when β=0	0	−0.019456	$4.45721\times 10^{-5}$	β=0.4	0	−0.003891	$8.91442\times 10^{-6}$
	0.2	−0.015565	$3.56577\times 10^{-5}$		0.2	−0.003113	$7.13154\times 10^{-6}$
	0.4	−0.011673	$2.67433\times 10^{-5}$		0.4	−0.002335	$5.34865\times 10^{-6}$
	0.6	−0.007782	$1.78288\times 10^{-5}$		0.6	−0.001556	$3.56577\times 10^{-6}$
	0.8	−0.003891	$8.91442\times 10^{-6}$		0.8	−0.000778	$1.78288\times 10^{-6}$
	1	0	0		1	0	*0*
Upper solution when β=1	0	0.019456	$4.45721\times 10^{-5}$	β=0.6	0	0.003891	$8.91442\times 10^{-6}$
	0.2	0.015565	$3.56577\times 10^{-5}$		0.2	0.003113	$7.13154\times 10^{-6}$
	0.4	0.011673	$2.67433\times 10^{-5}$		0.4	0.002335	$5.34865\times 10^{-6}$
	0.6	0.007782	$1.78288\times 10^{-5}$		0.6	0.001556	$3.56577\times 10^{-6}$
	0.8	0.003891	$8.91442\times 10^{-6}$		0.8	0.000778	$1.78288\times 10^{-6}$
	1	0	0		1	0	0

## Data Availability

Not applicable.
